# Community‐based health literacy focused intervention for cervical cancer control among Black women living with human immunodeficiency virus: A randomized pilot trial

**DOI:** 10.1111/hex.13644

**Published:** 2022-11-28

**Authors:** Hae‐Ra Han, Kyra J. W. Mendez, Nancy Perrin, Joycelyn Cudjoe, Gregory Taylor, Dorcas Baker, Jeanne Murphy‐Stone, Phyllis Sharps

**Affiliations:** ^1^ The Johns Hopkins University School of Nursing Baltimore Maryland USA; ^2^ The Johns Hopkins University Bloomberg School of Public Health Baltimore Maryland USA; ^3^ Center for Community Programs Innovation, and Scholarship Baltimore Maryland USA; ^4^ National Cancer Institute Bethesda Maryland USA; ^5^ United States Government Accountability Office District of Columbia Washington USA; ^6^ University of Maryland School of Medicine Baltimore Maryland USA; ^7^ Older Women Embracing Life (OWEL) Baltimore Maryland USA; ^8^ School of Nursing The George Washington University Washington DC USA

**Keywords:** cervical cancer screening, health literacy, human immunodeficiency virus, pilot, randomized trial

## Abstract

**Background:**

Health literacy plays an essential role in how individuals process health information to make decisions about health behaviours including cancer screening. Research is scarce to address health literacy as a strategy to improve cancer screening participation among women living with human immunodeficiency virus (HIV), particularly Black women who, despite the heavy burden of cervical cancer, report consistently low screening rates.

**Aim:**

To assess the feasibility, acceptability and preliminary efficacy of a health literacy‐focused intervention called CHECC‐uP—Community‐based, HEalth literacy focused intervention for Cervical Cancer control—among women living with HIV.

**Methods:**

We conducted a community‐based, single‐blinded randomized pilot trial. A total of 123 eligible women were enrolled and randomized to one of two conditions, control (i.e., cervical cancer brochure) or intervention (cervical cancer brochure plus 30–60 min health literacy‐focused education followed by monthly phone counselling and navigation assistance for 6 months). Study assessments were done at baseline, 3 and 6 months. The final analysis sample included 58 women who completed all data points and whose Papanicolaou (Pap) test status was confirmed by medical records.

**Results:**

All intervention participants who completed the programme would recommend the CHECC‐uP to other women living with HIV. However, adherence in the experimental conditions was low (49.6% attrition rate including 20 women who dropped out before the intervention began) due, in large part, to phone disconnection. Those who had received the intervention had a significantly higher Pap test rate compared to women in the control group at 6 months (50% vs. 21.9%, *p* = .025). Participation in the intervention programme was associated with improved health literacy and other psychosocial outcomes at 3 months but the trend was attenuated at 6 months.

**Conclusions:**

The CHECC‐uP was highly acceptable and led to improved Pap testing rates among Black women living with HIV. Future research should consider addressing social determinants of health such as phone connectivity as part of designing a retention plan targeting low‐income Black women living with HIV.

**Implications:**

The findings should be incorporated into a future intervention framework to fulfil the unmet needs of Black women living with HIV to facilitate their decision‐making about Pap test screening.

**Patient or Public Contribution:**

Nineteen community members including women living with HIV along with HIV advocates and care providers participated in four focus groups to develop cervical cancer screening decision‐relevant information and the health literacy intervention. Additionally, a community advisory board was involved to provide guidance in the general design and conduct of the study.

## 1 INTRODUCTION

Despite considerable progress in US cancer control over the past decades, certain groups continue to experience significant health disparities. Women living with human immunodeficiency virus (HIV) (WLH) experience a disproportionate cervical cancer burden because of an impaired immune response to the human papillomavirus, the virus that causes cervical cancer.^1^ In particular, Black women have the highest cervical cancer mortality.^2^ Regular Papanicolaou (Pap) testing is accepted as a critical strategy in the early detection and timely treatment of cervical cancer and precancerous lesions.^3^ Yet, a large cross‐sectional study found that cervical cancer screening decreased in the United States between 2005 and 2019.^4^ WLH, especially Black women have reported consistently lower Pap test rates compared to other groups.^5^

Health literacy—‘the degree to which individuals have the capacity to obtain, process, and understand basic health information and services to make appropriate health decisions’ (para. 1)—is a key social determinant of health and is recognized as an essential element of access to high‐quality, patient‐centred care.^6^, ^7^ Health literacy deficits are a significant barrier to obtaining Pap tests.^6^ While research on health literacy among WLH is scarce,^7^ studies involving women without HIV^8^, ^9^, ^10^ have reported that women with limited health literacy are more likely to misunderstand health information provided and find it difficult to convert and interpret proportions of their cancer risk, which increases women's misperceptions and lowers their personalization of such risks. Consequently, low health literacy negatively affects knowledge, attitudes and self‐efficacy with regard to cervical cancer screening.^8^, ^11^ Approximately 25%–38% of people living with HIV have limited health literacy,^12^, ^13^ compared to the national rate of 9% for the general US population.^14^ The rate of low health literacy is even higher among Black WLH. For example, in a recent cross‐sectional study, nearly half (49.6%) of the Black WLH had a reading level at or below sixth grade, suggesting that the women may struggle with most written health information.^15^

Systematic reviews and meta‐analyses^16^, ^17^ of interventions designed to increase Pap screening participation among ethnic minority populations revealed that interventions have focused primarily on increasing knowledge (e.g., causes, risk factors or signs and symptoms of the disease) or accommodating women's needs and have produced small effect sizes of 5%–24%. None of the studies in these reviews has attempted to directly address study participants' health literacy deficits as a strategy to improve cancer screening participation rates. Examples of health literacy interventions may include training on how and when to access healthcare, medical terminology training or numeracy training by using visual aids.^18^ Further, only one study addressed WLH, in which a randomized controlled trial was conducted to test an intervention where WLH collected their own human papillomavirus samples and then received counselling based on their results.^19^ This intervention failed to improve Pap test screening among WLH.^19^ There is a need for promising innovations that can address the health literacy needs of WLH, who suffer disproportionately from unequal cervical cancer burden.^1^, ^2^

The current study was designed to address this gap by testing a health literacy‐focused intervention programme called CHECC‐uP—Community‐based, HEalth literacy focused intervention for Cervical Cancer control among WLH. We conducted a pilot study with 3‐ and 6‐month follow‐ups to evaluate the feasibility, acceptability and preliminary efficacy of this intervention. We hypothesized that participation in the CHECC‐uP intervention would be associated with an increase in Pap testing and improvements in psychosocial outcomes.

## 2 METHODS

### 2.1 Design and sample

We used a community‐based, randomized controlled trial design to pilot test the CHECC‐uP intervention compared to an educational control (Clinical Trials Registry NCT03033888). Women were recruited from inner‐city HIV clinics, community organizations serving people with HIV or a university‐based HIV/AIDS research centre in Baltimore, MD, by posting study flyers in these organizations or advertising the study through social media (e.g., Facebook, Craigslist) and attending health fairs.^20^ Additionally, the study team received names and contact information of potential study participants through a university‐based HIV/AIDS research centre hotline; people living with HIV would call the HIV/AIDS research centre for potential research participation and hotline staff would provide their information to the study team if they met the study eligibility criteria.^20^ Upon these self‐ or direct referrals, trained study staff screened potential study participants for eligibility over the phone and scheduled a study visit for informed consent and baseline data collection. Eligible participants were: (1) women aged 18 years or older; (2) diagnosed with HIV; (3) overdue for a Pap test (e.g., no Pap test within the last 12 months at the time of study enrollment) and (4) could speak and understand English. Women with a hysterectomy were excluded. The study was designed to detect an increase of 20% in the proportion of women in the intervention arm completing Pap testing at 6‐month follow‐up, compared to those in the control arm, with 80% power and *α* of .05.^21^, ^22^ Assuming a drop‐out rate of 30%, we estimated that we would need to enroll a total of 122 women.

**Figure 1:**
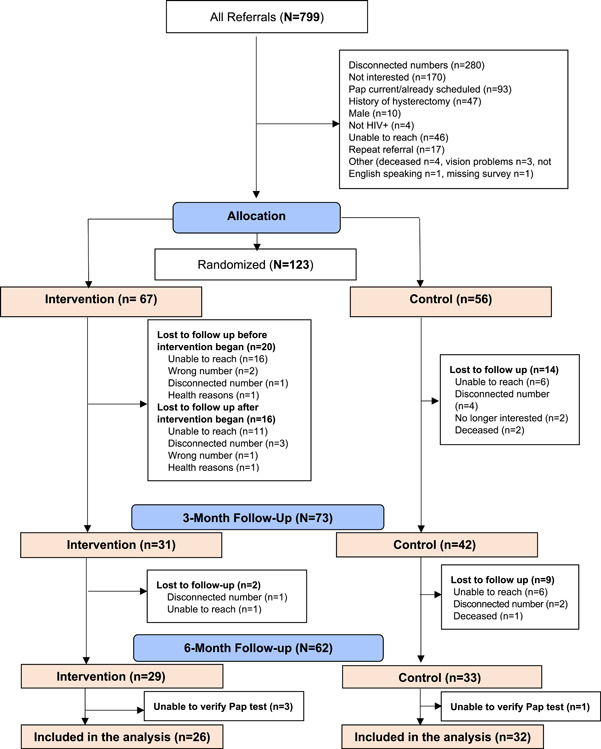
CONSORT

A total of 123 eligible women completed the study assessment at baseline and were randomized (intervention, *n* = 67; control, *n* = 56). Of those who completed the baseline assessment and were assigned to the intervention arm, 20 dropped out before the intervention began for several reasons including unable to reach after multiple attempts (*n* = 16), wrong or disconnected phone number (*n* = 3) and health reasons (*n* = 1). Those, whom we were unable to reach after attempting up to eight calls on different days and times (including weekends and evening hours), were considered dropouts. As a result, our intervention was delivered to 47 participants. Of those, 18 discontinued their participation before the final data collection assessments at 6 months were done, yielding 29 in the analysis sample for the intervention arm. As for the control arm, 23 dropped out over the course of the study, yielding 33 in the analysis sample (Figure 1).

### 2.2 Randomization and intervention

We used computer‐generated random numbers to randomize women to either the intervention or control arm. The control arm received an educational brochure related to cervical cancer among WLH created for the purpose of the study. Women in the intervention arm received the educational brochure plus the study intervention, which consisted of health literacy education and phone counselling with navigation assistance. Specifically, a trained community health worker delivered health literacy education at a community centre conveniently located near a subway station in the central downtown area. The health literacy education (Table 1) was designed to promote WLH's understanding of basic medical terminology used in cervical cancer screening; relevant medical instructions, such as appointment slips or follow‐up screening instructions and familiarity with how to navigate the healthcare system for Pap test screening.

**Table 1:** Main educational topics with examples of medical terminologies and role‐play contents

Topic	Example medical terminology practised	Example content covered in role‐play
HIV and cancer	Human papillomavirus, cancer, sexually transmitted disease	
What is Pap smear	Cervix, Pap smear	In the doctor's office: History taking
Why is it important to receive a Pap test	Cervical cancer, reproductive organs	
Cervical cancer symptoms	Hormones, genital warts	
How is Pap smear done	Laboratory (lab), pelvic exam, polyp, speculum	In the doctor's office: Pap smear
Things to remember before and after Pap smear	Abnormalities, history taking	

Abbreviations: HIV, human immunodeficiency virus; Pap, Papanicolaou.

Development of our intervention to include health literacy as its core component was guided by the Precede‐Proceed model,^23^ which identifies critical constructs as predisposing (e.g., individual characteristics), enabling (e.g., health literacy knowledge, self‐efficacy) and reinforcing factors (e.g., cultural beliefs and attitudes). HIV community advocates, Black WLH and clinicians working closely with WLH engaged in the formative work to develop the health literacy intervention by sharing their experiences at OB/GYN clinics and identifying common scenarios and dialogues that occur between the patient and medical staff when navigating a Pap test screening. Based on this formative work, a picture guidebook was created as educational material for WLH.

At the end of the health literacy‐focused education session, women in the intervention group received a copy of the picture guidebook to reinforce what they had learned and practised in class. The follow‐up portion of the study intervention included monthly phone counselling for up to 6 months. Using a checklist addressing key talking points, the objectives of the follow‐up were to (1) reinforce health literacy knowledge and skills learned and practised from the education session; (2) address any questions or concerns the participant might have and (3) provide tailored navigation assistance with individually identified barriers to Pap test screening over a 6‐month period.

### 2.3 Procedures

The Johns Hopkins Institutional Review Board approved the study protocol. Once eligible women were identified, trained research assistants scheduled a visit to obtain written informed consent and collect baseline data at several community sites (e.g., nurse‐run community health centres or community organizations serving people with HIV). Upon completion of the baseline assessment, a trained community health worker delivered health literacy education to women assigned to the intervention arm.

Initially, education sessions were scheduled for groups of six to eight women. However, the group‐based format presented substantial scheduling challenges to the study team with high rates of no‐shows. This led to the study team's decision to adopt individual education delivery. The education sessions lasted about 30 min, when offered individually, and 45–60 min, when offered as a group due to discussion during the group session. Within 1–2 weeks after completing education, intervention participants received monthly phone counselling sessions for 6 months. During each phone call, a counsellor checked the participant's progress toward completing a Pap test and answered questions or concerns about Pap test screening.

For both intervention and control arms, we provided a copy of the Pap test brochure tailored to WLH, highlighting causes and symptoms of cervical cancer, risk factors for cervical cancer among WLH, the value of Pap screening and how to prepare for a Pap test. All of our educational materials were written at a sixth grade level or lower, as assessed by Flesch‐Kincaid grade‐level statistics in Microsoft Word. Additionally, all women in the study received a list of local community resources where a Pap test could be obtained free, or at a reduced cost, based on a sliding scale.

Trained study staff who were blinded to the group assignment collected data at baseline, 3 and 6 months from the start of the intervention. After 6 months, intervention women were invited to postintervention qualitative interviews to share their experiences with CHECC‐uP. Every woman provided informed written consent. Enrolled participants received $20 at baseline and 3 months and $40 at 6 months for their time. Postintervention interview participants received an additional $30.

### 2.4 Measures

A study questionnaire was used to collect participants sociodemographic and medical characteristics. Data regarding Pap test status were assessed via medical record review. We used several study instruments to assess changes in WLH's psychosocial outcomes: Health literacy, cancer knowledge, self‐efficacy, cultural beliefs/attitudes and depression. We include the internal consistency for each instrument, which was calculated using the full sample (*n* = 123) at baseline.

To assess health literacy, we used familiarity, navigation and numeracy subscales from the Assessment of Health Literacy in Cancer Screening (AHL‐C), a validated comprehensive health literacy instrument with *α* coefficients ranging from .70 to .96.^24^ Building on Baker's conceptual model of health literacy,^25^ the AHL‐C addresses multiple types of health literacy in cancer screening, such as reading ability, familiarity, navigation, comprehension and numeracy. We chose familiarity, navigation and numeracy because they have been associated with cancer knowledge,^26^ risk perception,^27^ intent to get cancer screening^26^, ^28^ and actual cervical cancer screening behaviour.^29^ The familiarity subscale includes 12 items (5‐point Likert scale; 1 = never heard before to 5 = can use fluently) with scores ranging from 12 to 60. The navigation and numeracy subscales include 12 and 7 items, respectively; each correct response to the items on the subscales is coded as 1, with possible scores ranging from 0 to 12 and 0 to 7, respectively. Example questions included: ‘How familiar are you with the following words’ or ‘Please read the passages below and select a word to fill in each blank’. *α* Coefficients ranged from .51 to .94 in the study sample.

Cancer knowledge was measured by the Cervical Cancer Knowledge (CCK) Test which consists of 10 items (*α* coefficient = .80–.89).^30^ An example question is ‘If one smokes heavily, the risk for cervical cancer increases’. Given the direct link between HPV and cervical cancer, we added 12 items about HPV to the CCK Test (e.g., ‘A person who has HPV needs to have Pap smears more often than others’). Correct responses to each of the knowledge questions were scored 1, with possible total scores from 0 to 22. Higher scores indicated higher cancer knowledge. The modified CCK Test had an *α* coefficient of .77 in the study sample.

Self‐efficacy related to cervical cancer screening was assessed by the Cervical Cancer Self‐Efficacy scale.^31^ The self‐efficacy scale includes four items (4‐point Likert scale; 1 = not at all confident to 4 = very confident) asking how confident a woman is in carrying out tasks related to Pap tests, with higher scores indicating higher self‐efficacy. An example question is ‘Do you feel confident that you can schedule a Pap test appointment and keep it?’ The scale was validated in Mexican and Korean American women with high internal consistency reliability coefficients ranging from 0.92 to 0.95.^32^, ^33^ The *α* coefficient was .89 in this sample.

Cultural beliefs and attitudes were assessed using a modified nine‐item inventory (5‐point Likert; 1 = strongly disagree to 5 = strongly agree), which was adopted from the cultural barriers to breast and cervical cancer screening questionnaire.^34^, ^35^ The original scale was validated on young Asian American women and older Chinese American women with *α* coefficients ranging from .61 to .72. Example questions include ‘I would feel embarrassed with a doctor examining my cervix as part of a medical exam’, and ‘I only see a doctor when I am having a health problem’. The *α* coefficient of the modified scale was .8 in the study sample.

Depressive symptoms were measured using the Patient Health Questionnaire‐9 (PHQ‐9). PHQ‐9 is a well‐validated and widely disseminated screener for depressive symptoms.^36^ The score of each participant is calculated by summing the scores for nine questions (4‐point Likert scale; 0 = not at all to 3 = nearly every day) asking about the presence of signs and symptoms of depression during the 2 weeks before the survey. Total scores range from 0 to 27, with scores of 5, 10, 15 and 20 represent mild, moderate, moderately severe and severe depression, respectively. The Cronbach's *α* of the PHQ‐9 was .88 in the study sample.

We also collected data on the feasibility and acceptability of the CHECC‐uP. The feasibility of the study was examined using multiple sources of data, such as study recruitment and retention, attendance at education sessions and follow‐up phone counselling completion rates. Acceptability was assessed using a questionnaire developed for the purpose of this study. The survey included self‐reported satisfaction with the intervention programme, as well as the receipt (e.g., reading the intervention material), helpfulness and application (e.g., applied contents from the material to get a Pap test) of intervention materials.

### 2.5 Statistical analyses

Analysis was performed using data from the 58 participants who completed all data points and whose Pap test status was confirmed objectively by medical records (Figure 1). We used descriptive statistics such as means, standard deviations (SDs) and frequencies to establish analysis sample characteristics and study variables. Intervention and control groups were compared at baseline using chi‐squared tests or independent sample *t*‐tests. The primary efficacy outcome was the completion of a Pap test, which was tested with a *χ* ^2^ test. Change over time in the psychosocial outcomes was tested with repeated measures analysis of variance with time, group and the group × time interactions included in the model. We calculated effect sizes using the group difference in the mean change from baseline to 3‐month follow‐up, and the group difference in the mean change from baseline to 6 months follow‐up, each divided by the baseline SD.^37^

## 3 RESULTS

### 3.1 Sample characteristics

The final sample size included 58 participants (Figure 1). There were significant differences in age and cultural beliefs and attitudes scores between the participants who completed the study and those who did not. Specifically, participants who completed the study were 4 years older (*p* = .008) and had a 2.3‐point lower score on the cultural beliefs and attitudes scale (*p* = .048) at baseline. Among the intervention group women, there were no significant differences between participants who did not complete the intervention (*n* = 38) and those who did (*n* = 29).

The baseline characteristics of 58 participants included in the analysis are summarized in Table 2. The only significant difference between intervention and control groups at baseline was that participants in the intervention group were about 6 years younger, on average than participants in the control group (*p* = .003). Overall, the participants in the analysis sample were middle‐aged (mean: 53.5 years, SD: 7.8) and all were Black or African American. Most women were never married (49.1%), separated, widowed or divorced (29.8%). More than 40% of women had less than a high school education. Nearly 9 out of 10 (89.5%) were unemployed, retired or disabled, and only 27.3% of WLH reported they could live comfortably or very comfortably with their income. The majority of our sample was renting their current residence (69%). Finally, most had a primary care physician (98.2%) and 96.4% of women reported having a pap test at some time in their life.

**Table 2:** Analysis sample characteristics at baseline (*N* = 58)

Variable	Total (*N* = 58), *n* (%) or mean ± SD	Control (*n* = 32), *n* (%) or mean ± SD	Intervention (*n* = 26), *n* (%) or mean ± SD	*p* Value
Age in years (range = 28–67)	53.5 ± 7.8	56.3 ± 5.6	49.8 ± 8.9	.003
Black/African American	58 (100)	32 (100)	26 (100)	
Marital status				.053
Married or partnered	12 (21.3)	3 (9.4)	9 (36.0)	
Separated, widowed or divorced	17 (29.8)	13 (40.6)	4 (16.0)	
Never married	28 (49.1)	16 (50.0)	12 (48.0)	
Missing	1		1	
Education				.246
<High school	24 (42.1)	14 (45.2)	10 (38.5)	
High school	18 (31.6)	7 (22.6)	11 (42.3)	
Some college or more	15 (26.3)	10 (32.3)	5 (19.2)	
Missing	1	1		
Employment				.820
Working full‐ or part‐time	6 (10.5)	3 (9.7)	3 (11.5)	
Unemployed, retired or disabled	51 (89.5)	28 (90.3)	23 (88.5)	
Missing	1	1		
Income level				.826
Very comfortable or comfortable	15 (27.3)	9 (29.0)	6 (25.0)	
Just OK	24 (43.6)	14 (45.2)	10 (41.7)	
Difficult/very difficult to manage	16 (29.1)	8 (25.8)	8 (33.3)	
Missing	3	1	2	
Type of residence				.957
Own	3 (5.2)	2 (6.3)	1 (3.8)	
Renting	40 (69.0)	22 (68.8)	18 (69.2)	
Public housing	10 (17.2)	5 (15.6)	5 (19.2)	
Other	5 (8.6)	3 (9.4)	2 (7.7)	
Have health insurance	58 (100)	32 (100)	26 (100)	
Have PCP	56 (98.2)	31 (96.9)	25 (96.2)	.373
Ever had a Pap test (Yes)	54 (96.4)	31 (96.9)	23 (96.9)	.109
Own a smartphone	37 (77.1)	18 (72.0)	19 (82.6)	.382

Abbreviations: Pap, Papanicolaou; PCP, primary care provider.

### 3.2 Feasibility and acceptability

We recruited and randomized the target sample size of 123 with a retention rate of 50.4% (or 60.2% after accounting for early dropouts in the intervention arm; e.g., those who completed the baseline assessment but left the study before receiving the study intervention, *n* = 20). The size of health literacy education classes ranged from 1 to 5 participants before we changed it into an individually based format. The intervention participants in the analysis sample completed on average about one phone counselling session (range = 0–4; median = 1).

The CHECC‐uP intervention was highly acceptable. All intervention women who responded to the acceptability questionnaire (*n* = 26) would recommend the programme to other WLH. Nearly all respondents to the questionnaire were satisfied or very satisfied with the information they learned about Pap test screening (96.2%) and the way they learned (96.2%). More than two thirds (73.1%) of the responders indicated they read the picture guidebook on their own, partially or entirely. Slightly more than a quarter of them (26.3%) used the picture guidebook when getting a Pap test to better understand the process. Eighty percent of women who used the picture guidebook found it helpful and 20% somewhat helpful. Similarly, the majority of responders reported that they read the Pap test brochure and the community resource list partially or entirely (88.5% and 76.9%, respectively). Of those who read the brochure, 87% found it helpful or very helpful. Forty percent (40%) of women who read the community resource list indicated that they used the list to find a place for a Pap test; 75% noted the list as being very helpful or helpful and 25% somewhat helpful.

### 3.3 Changes in Pap test screening and psychosocial outcomes

At 6 months, 50% of WLH in the intervention group received a Pap test, compared to 21.9% of women in the control group (28.1% difference; *χ* ^2^ = 5.02, *p* = .025). The effect sizes of the CHECC‐uP intervention on psychosocial outcomes at 3 and 6 months are presented in Table 3. At baseline, the overall analysis sample had relatively high levels of familiarity (mean = 39.5, SD = 13.2) and health navigational literacy (mean = 10.9, SD = 1.76) and low levels of numeracy (mean = 3.44, SD = 1.62). At 3 months, the mean increases in familiarity and numeracy were greater in WLH in the intervention arm compared to those in the control arm with effect sizes of 0.34 and 0.23, respectively. The mean increase in health navigational literacy was higher in WLH in the control arm at 3 months, but the trend reversed favouring the intervention arm at 6 months with a negligible effect size.

**Table 3:** Outcome changes over 6 months^a^

Variable	Mean						SD at baseline	Effect size at 3 months^b^	Effect size at 6 months^c^
	Baseline		3 months		6 months				
	Control	Intervention	Control	Intervention	Control	Intervention			
*Familiarity* (possible range = 12–60)	39.11	39.81	38.16	43.44	43.28	46.96	13.37	0.34 (*p* = .068)	0.34 (*p* = .185)
*Navigation* (possible range = 0–12)	10.88	10.74	10.98	10.44	11.00	10.96	1.78	−0.23 (*p* = .439)	0.09 (*p* = .731)
*Numeracy* (possible range = 0–7)	3.32	3.15	4.00	4.19	3.56	3.65	1.58	0.23 (*p* = .332)	0.23 (*p* = .403)
Knowledge (possible range = 0–22)	9.98	10.33	11.33	12.81	11.56	12.38	3.99	0.28 (*p* = .203)	−0.05 (*p* = .826)
Self‐efficacy (possible range = 4–16)	14.59	14.73	14.31	15.04	14.68	15.5	2.32	0.25 (*p* = .423)	0.19 (*p* = .494)
Cultural beliefs (possible range = 9–45)	15.92	17.21	15.55	13.50	16.44	14.08	5.75	−0.58 (*p* = .024)	−0.54 (*p* = .065)
Depression (possible range = 0–27)	14.94	15.75	14.39	13.65	12.91	14.65	5.53	−0.28 (*p* = .349)	<−0.01 (*p* = .998)

*Note*: Health literacy variables are italicized.

Abbreviation: Pap, Papanicolaou.

^a^ *n* = 58 with full data on both psychosocial outcomes and Pap test status based on medical record review.

^b^ Group difference in mean change scores from baseline to 3 months divided by the standard deviation at baseline.

^c^ Group difference in mean change scores from baseline to 6 months divided by the standard deviation at baseline.

For other psychosocial variables, the absolute value of effect sizes ranged from 0.25 to 0.58 at 3 months and from <0.01 to 0.54 at 6 months. For cervical cancer knowledge, the intervention arm had a greater increase at 3 months, but the difference was not sustained at 6 months, with relatively no difference between the intervention and control arms. For self‐efficacy, the intervention arm had a greater increase, but with a reduced effect size at 6 months. Cultural beliefs addressing cultural barriers, such as modesty, declined for both groups at 3 months, with the intervention arm having a significantly greater reduction. At 6 months, the intervention arm maintained the declining trend while the control arm reversed back. Finally, depression scores declined for both groups at 3 months, but the intervention arm had a greater decrease with an effect size of 0.28; though the difference was not sustained at 6 months. For all psychosocial variables, the statistical test of change over time was significant only for cultural beliefs at 3 months (*p* = .024).

## 4 DISCUSSION

We found that a multifaceted, health literacy‐focused intervention (CHECC‐uP) can promote Pap testing among Black WLH. However, we experienced a high attrition rate in the study sample. The findings demonstrate the preliminary efficacy of CHECC‐uP for Black WLH as a potential strategy to reduce cervical cancer disparities in this population. To the best of our knowledge, CHECC‐uP is the first intervention to integrate health literacy education as an active component to promote Pap testing among WLH. The statistically significant difference in Pap test rates observed among WLH in the trial (28.1% difference) is higher than other reported rates for HIV‐negative women, ranging from 5% to 24%.^16^, ^17^ The theory‐driven intervention programme was well received by our sample, as evidenced by the acceptability measures including 100% of intervention women in the analysis sample who would recommend the CHECC‐uP to other WLH. We believe the involvement of community stakeholders in developing the intervention approach may have helped to promote the credibility of CHECC‐uP as relevant to the target community.^38^

Health literacy consists of multiple dimensions that go beyond one's reading ability.^29^ Of the three dimensions measured in the study, the effect sizes for both familiarity and numeracy favoured the intervention and remained consistent at 3 and 6 months. In contrast, the effect size for navigational health literacy was either not in favour of the intervention arm, or negligible. Navigational health literacy addresses one's understanding of how to navigate the process of undergoing cancer screening (e.g., check‐in and ‐out at an OB/GYN clinic, dialogue between a woman and a doctor about risk factors for cervical cancer).^24^ Our finding may be a result of the study sample mostly being recruited from HIV clinics or an HIV/AIDS research centre (65.9%).^20^ Different from prior research, in which women without HIV were recruited from nonclinical settings such as ethnic churches, the current study sample included women with prior exposure to the healthcare system. Nearly perfect baseline scores on the navigation subscale (possible ranges = 0–12) observed in both the intervention and control arms (about 11 points) indicate a high ceiling effect with the limited utility of the subscale as a health literacy outcome measure in our sample of WLH.

Our retention rate was not optimal. We had higher dropouts among younger women and women who scored higher on the cultural attitudes and beliefs scale. The role of age in cervical cancer screening participation is not at all consistent.^39^, ^40^, ^41^ Cultural beliefs and attitudes in cancer screening address embarrassment about the body or sexuality and modesty.^34^, ^35^ A recent focus group study involving WLH noted feelings of shame and embarrassment when talking about cervical cancer and Pap smears as a barrier to screening for WLH.^38^ These findings suggest the need for more tailored retention approaches to those at risk for dropout by showing empathy, active listening and open communication to allow expressing one's feeling, while also sharing acceptable strategies based on beliefs (e.g., community resources listing clinics with female doctors).^42^ Additionally, a recent review of the literature for recruitment and retention of WLH in clinical studies reported attrition rates between 15% and 33%.^20^ The published studies included in the review used on‐site staff and/or multiple engagement methods to retain participants (e.g., sending holiday or birthday cards, sending newsletters or offering stipends for childcare or transportation to study sites).^20^ Due to constraints in terms of resources, our study used trained study staff to recruit women from participating sites, upon referrals, with the main methods of engagement being reminder calls and nominal stipends for transportation to the data collection sites. The findings highlight the need for working with HIV clinical partners and the use of multiple, individually tailored engagement approaches to retain WLH in a clinical trial.

Another important lesson learned from this pilot trial is that at least one‐third of the attrition observed in our study was early dropouts in the intervention arm, which led to a change in the education format from group sessions to individually based sessions. Benefits of group‐based education have included cost saving^43^ and peer support.^44^ Despite our best efforts, the study team experienced logistical challenges in scheduling group education sessions (with delays of up to a month or longer) due to the different schedules and needs of WLH. The challenges were due, in large part, to phone disconnection. According to a national report,^45^ adults living in poverty (69%) or in rented homes (76%) had a higher probability of being ‘wireless only’, with no landline telephone, compared to higher‐income adults (59%) and adults living in a house owned by a household member (53%; p. 3). Nevertheless, physical access to cell phones may not be enough to ensure connectivity. Adults with low incomes often must purchase minutes because they do not have cell phone plans.^46^ Without contract plans, users often must change numbers, or get disconnected, until more minutes are purchased, resulting in people experiencing periods of ‘phonelessness’ (p. 1428).^46^ Future trials involving low‐income WLH should consider addressing phone connectivity as part of their retention plan. For example, the federal Lifeline programme provides discounted or free phones and services for low‐income families in the United States.^47^

Study limitations include an insufficient sample size to detect a statistically significant change in outcomes, which resulted from high attrition. Nevertheless, the effect sizes estimated for the study variables are encouraging and warrant further investigation to test the efficacy of the intervention, especially given the high acceptability and satisfaction of WLH with the study intervention. Additionally, given the multifaceted nature of the study intervention, we are unable to tease out active intervention components. The intervention acceptability indicators (e.g., satisfaction with the intervention and receipt, helpfulness and application of the intervention materials) seem to suggest overall synergy between health literacy education and follow‐up components, which should be maintained in future implementations of CHECC‐uP. Finally, the generalizability of study findings is limited by the inclusion of only Black, African American women in the study sample from a low‐income, urban community. Nationally, 41% of WLH are Black, with 49% having less than a high school education and 44% having household incomes at or below federal poverty guidelines.^48^ In Baltimore, 72% of WLH are Black, 35%–55% have less than a high school level of education, and about 64% have low‐income status.^49^, ^50^ Future research should include more diverse groups of WLH from different cultural and racial/ethnic backgrounds.

## 5 CONCLUSION

Pilot testing of the CHECC‐uP intervention resulted in promising effect sizes and high acceptability among low‐income Black WLH. We incorporated health literacy education as a new approach to promote Pap test screening among WLH. The findings support integrating health literacy into a future intervention framework to transform the design of cervical cancer screening interventions for WLH. High attrition observed in our study sample highlights the need for considering systematic strategies, such as the federal Lifeline programme for free phones and services, in future trials to successfully retain a study sample from underserved, low‐income communities. It is possible that the positive effects of improved health literacy required for the uptake of cervical cancer screening may be more evident with a larger sample size than that of our pilot trial.

## AUTHOR CONTRIBUTIONS

All authors approved the final version of the manuscript. Hae‐Ra Han originated the study and led the writing. Jeanne Murphy‐Stone and Phyllis Sharps contributed to the development of the study concept and design. Hae‐Ra Han, Kyra J. W. Mendez, Nancy Perrin, Joycelyn Cudjoe, Gregory Taylor and Dorcas Baker contributed to the acquisition, analysis or interpretation of data. Hae‐Ra Han drafted the manuscript, and all authors contributed to the critical revision of the manuscript. Hae‐Ra Han also supervised the study.

## CONFLICT OF INTEREST

The authors declare no conflict of interest.

## ETHICS STATEMENT

The study was approved by the Johns Hopkins Medicine IRB. Informed consent was obtained from all individual participants included in the study.

## Data Availability

Study data will be made available upon reasonable request to the corresponding author. De‐identified data will be made available upon reasonable request.

## References

[1] Castle PE , Einstein MH , Sahasrabuddhe VV . Cervical cancer prevention and control in women living with human immunodeficiency virus. CA Cancer J Clin. 2021;71(6):505‐526. 10.3322/caac.21696 34499351 PMC10054840

[2] National Cancer Institute . Cancer stat facts: Cervical cancer. Accessed October 31, 2022. https://seer.cancer.gov/statfacts/html/cervix.html

[3] Clinical Info HIV.gov . Guidelines for the prevention and treatment of opportunistic infections in adults and adolescents with HIV. Accessed October 31, 2022. https://clinicalinfo.hiv.gov/en/guidelines/adult-and-adolescent-opportunistic-infection/human-papillomavirus-disease

[4] Suk R , Hong YR , Rajan SS , Xie Z , Zhu Y , Spencer JC . Assessment of US preventive services task force guideline‐concordant cervical cancer screening rates and reasons for underscreening by age, race and ethnicity, sexual orientation, rurality, and insurance, 2005 to 2019. JAMA Netw Open. 2022;5:e2143582. 10.1001/jamanetworkopen.2021.43582 35040970 PMC8767443

[5] Fletcher FE , Vidrine DJ , Tami‐Maury I , et al. Cervical cancer screening adherence among HIV‐positive female smokers from a comprehensive HIV clinic. AIDS Behav. 2014;18(3):544‐554. 10.1007/s10461-013-0480-6 23605155 PMC4383031

[6] Health Resources and Services Administration . Health literacy. Accessed October 31, 2022. https://www.hrsa.gov/about/organization/bureaus/ohe/health-literacy/index.html

[7] Budhwani H , Gakumo CA , Yigit I , et al. Patient health literacy and communication with providers among women living with HIV: a mixed methods study. AIDS Behav. 2022;26(5):1422‐1430. 10.1007/s10461-021-03496-2 34642834 PMC9001740

[8] Lindau ST , Tomori C , Lyons T , Langseth L , Bennett CL , Garcia P . The association of health literacy with cervical cancer prevention knowledge and health behaviors in a multiethnic cohort of women. Am J Obstet Gynecol. 2002;186(5):938‐943. 10.1067/mob.2002.122091 12015518

[9] Kassaman D , Mushani T , Kiraithe P , Brownie S , Barton‐Burke M . Fear, faith and finances: health literacy experiences of English and Swahili speaking women newly diagnosed with breast and cervical cancer. Ecancermedicalscience. 2022;16:1350. 10.3332/ecancer.2022.1350 35242231 PMC8831107

[10] Rutherford EJ , Kelly J , Lehane EA , et al. Health literacy and the perception of risk in a breast cancer family history clinic. Surgeon. 2018;16(2):82‐88. 10.1016/j.surge.2016.06.003 27908542

[11] Scott TL , Gazmararian JA , Williams MV , Baker DW . Health literacy and preventive health care use among Medicare enrollees in a managed care organization. Med Care. 2002;40(5):395‐404. 10.1097/00005650-200205000-00005 11961474

[12] Hicks G , Barragan M , Franco‐Paredes C , Williams MV , del Rio C . Health literacy is a predictor of HIV/AIDS knowledge. Fam Med. 2006;38(10):717‐723. 17075745

[13] Drainoni ML , Rajabiun S , Rumptz M , et al. Health literacy of HIV‐positive individuals enrolled in an outreach intervention: results of a cross‐site analysis. J Health Commun. 2008;13(3):287‐302. 10.1080/10810730801985442 18569359

[14] U.S. Department of Education . The health literacy of America's adults: results from the 2003 national assessment of adult literacy. 2006. Accessed September 2, 2021. http://nces.ed.gov/pubs2006/2006483.pdf

[15] Sun CA , Chepkorir J , Mendez KJW , Cudjoe J , Han HR . A descriptive analysis of cancer screening health literacy among Black women living with HIV in Baltimore, Maryland. Health Lit Res Pract. 2022;6(3):e175‐e181. 35858186 10.3928/24748307-20220616-01 PMC9272572

[16] Han HR , Kim J , Lee JE , et al. Interventions that increase use of pap tests among ethnic minority women: a meta‐analysis. Psychooncology. 2011;20(4):341‐351. 10.1002/pon.1754 20878847 PMC3741532

[17] Chan DNS , So WKW . A systematic review of randomised controlled trials examining the effectiveness of breast and cervical cancer screening interventions for ethnic minority women. Eur J Oncol Nurs. 2015;19(5):536‐553. 10.1016/j.ejon.2015.02.015 25840817

[18] Visscher BB , Steunenberg B , Heijmans M , et al. Evidence on the effectiveness of health literacy interventions in the EU: a systematic review. BMC Public Health. 2018;18(1):1414. 30594180 10.1186/s12889-018-6331-7 PMC6310940

[19] Murphy J , Mark H , Anderson J , Farley J , Allen J . A randomized trial of human papillomavirus self‐sampling as an intervention to promote cervical cancer screening among women with HIV. J Low Genit Tract Dis. 2016;20(2):139‐144. 10.1097/LGT.0000000000000195 27015260 PMC4808515

[20] Mendez KJW , Cudjoe J , Strohmayer S , Han HR . Recruitment and retention of women living with HIV for clinical research: a review. AIDS Behav. 2021;25(10):3267‐3278. 10.1007/s10461-021-03273-1 33990902 PMC8419017

[21] Lam TK , McPhee SJ , Mock J , et al. Encouraging Vietnamese‐American women to obtain pap tests through lay health worker outreach and media education. J Gen Intern Med. 2003;18(7):516‐524. 10.1046/j.1525-1497.2003.21043.x 12848834 PMC1494888

[22] Taylor VM , Hislop TG , Jackson JC , et al. A randomized controlled trial of interventions to promote cervical cancer screening among Chinese women in North America. J Natl Cancer Inst. 2002;94(9):670‐677. 11983755 10.1093/jnci/94.9.670 PMC1592333

[23] Green LW . Health Program Planning: An Educational and Ecological Approach; 2004. McGraw‐Hill.

[24] Han HR , Huh B , Kim MT , Kim J , Nguyen T . Development and validation of the assessment of health literacy in breast and cervical cancer screening. J Health Commun. 2014;19:267‐284. 10.1080/10810730.2014.936569 25315598 PMC4751992

[25] Baker DW . The meaning and the measure of health literacy. J Gen Intern Med. 2006;21(8):878‐883. 10.1111/j.1525-1497.2006.00540.x 16881951 PMC1831571

[26] Smith SG , Kobayashi LC , Wolf MS , Raine R , Wardle J , von Wagner C . The associations between objective numeracy and colorectal cancer screening knowledge, attitudes and defensive processing in a deprived community sample. J Health Psychol. 2016;21(8):1665‐1675. 10.1177/1359105314560919 25512199

[27] Koo K , Brackett CD , Eisenberg EH , Kieffer KA , Hyams ES . Impact of numeracy on understanding of prostate cancer risk reduction in PSA screening. PLoS One. 2017;12(12):e0190357. 10.1371/journal.pone.0190357 29284055 PMC5746255

[28] Schwartz PH , Perkins SM , Schmidt KK , Muriello PF , Althouse S , Rawl SM . Providing quantitative information and a nudge to undergo stool testing in a colorectal cancer screening decision aid: a randomized clinical trial. Med Decis Making. 2017;37(6):688‐702. 10.1177/0272989X17698678 28398836

[29] Han HR , Kim K , Cudjoe J , Kim MT . Familiarity, navigation, and comprehension: key dimensions of health literacy in pap test use among Korean American women. J Health Commun. 2019;24(6):585‐591. 10.1080/10810730.2019.1607955 31046641 PMC6803056

[30] Park S , Chang S , Chung C . Effects of a cognition‐emotion focused program to increase public participation in Papanicolaou smear screening. Public Health Nurs. 2005;22(4):289‐298. 10.1111/j.0737-1209.2005.220404.x 16150010

[31] Hogenmiller JR , Atwood JR , Lindsey AM , Johnson DR , Hertzog M , Scott JC Jr. Self‐efficacy scale for pap smear screening participation in sheltered women. Nurs Res. 2007;56(6):369‐377. 10.1097/01.NNR.0000299848.21935.8d 18004183

[32] Fernández E , Diamond PM , Rakowski W , et al. Development and validation of a cervical cancer screening self‐efficacy scale for low‐income Mexican American women. Cancer Epidemiol Biomarkers Prevent. 2009;18(3):866‐875. 10.1158/1055-9965.EPI-07-2950 PMC3062501 19258484

[33] Kim K , Xue QL , Walton‐Moss B , Nolan MT , Han HR . Decisional balance and self‐efficacy mediate the association among provider advice, health literacy and cervical cancer screening. Eur J Oncol Nurs. 2018;32:55‐62. 10.1016/j.ejon.2017.12.001 29353633 PMC6984402

[34] Tang TS , Solomon LJ , Yeh CJ , Worden JK . The role of cultural variables in breast self‐examination and cervical cancer screening behavior in young Asian women living in the United States. J Behav Med. 1999;22(5):419‐436. 10.1023/a:1018653306776 10586380

[35] Tang TS , Solomon LJ , McCracken LM . Cultural barriers to mammography, clinical breast exam, and breast self‐exam among Chinese‐American women 60 and older. Prev Med. 2000;31(5):575‐583. 10.1006/pmed.2000.0753 11071839

[36] Kroenke K , Spitzer RL , Williams JBW . The PHQ‐9: validity of a brief depression severity measure. J Gen Intern Med. 2001;16(9):606‐613. 10.1046/j.1525-1497.2001.016009606.x 11556941 PMC1495268

[37] Lichstein KL , Riedel BW , Grieve R . Fair tests of clinical trials: a treatment implementation model. Adv Behav Res Ther. 1994;16(1):1‐29.

[38] Jin S , Cudjoe J , Peay A , et al. Barriers and facilitators of pap testing for women living with HIV: a focus group study. J Assoc Nurses AIDS Care. 2020;31(2):190‐196. 10.1097/JNC.0000000000000126 31567730 PMC7047622

[39] Marques P , Nunes M , Antunes ML , Heleno B , Dias S . Factors associated with cervical cancer screening participation among migrant women in Europe: a scoping review. Int J Equity Health. 2020;19(1):160. 32917224 10.1186/s12939-020-01275-4 PMC7488650

[40] Jillapalli R , Radhakrishnan K . Cervical cancer screening behaviors among Asian Indians in the United States: a systematic review. J Immigr Minor Health. 2022;24:779‐789. 34273046 10.1007/s10903-021-01237-0

[41] Chua B , Ma V , Asjes C , Lim A , Mohseni M , Wee HL . Barriers to and facilitators of cervical cancer screening among women in Southeast Asia: a systematic review. Int J Environ Res Public Health. 2021;18:4586. 33926019 10.3390/ijerph18094586 PMC8123618

[42] Andrews CS . Modesty and healthcare for women: understanding cultural sensitivities. Community Oncol. 2006;3(7):443‐446.

[43] Molsted S , Tribler J , Poulsen PB , Snorgaard O . The effects and costs of a group‐based education programme for self‐management of patients with type 2 diabetes. A community‐based study. Health Educ Res. 2012;27(5):804‐813. 10.1093/her/cyr053 21712503

[44] Odgers‐Jewell K , Ball LE , Kelly JT , Isenring EA , Reidlinger DP , Thomas R . Effectiveness of group‐based self‐management education for individuals with type 2 diabetes: a systematic review with meta‐analyses and meta‐regression. Diabetic Med. 2017;34(8):1027‐1039. 10.1111/dme.13340 28226200

[45] Blumberg SJ , Luke JV . Wireless substitution: early release of estimates from the National Health Interview Survey, July–December 2019. 2020. https://www.cdc.gov/nchs/data/nhis/earlyrelease/wireless202009-508.pdf

[46] Gonzales AL , Ems L , Suri VR . Cell phone disconnection disrupts access to healthcare and health resources: a technology maintenance perspective. New Media Soc. 2016;18(8):1422‐1438. 10.1177/1461444814558670

[47] Federal Communications Commission . Lifeline program for low‐income consumers. Accessed October 31, 2022. https://www.fcc.gov/general/lifeline-program-low-income-consumers

[48] Blair JM , Fagan JL , Frazier EL , et al. Behavioral and clinical characteristics of persons receiving medical care for HIV infection—Medical Monitoring Project, United States, 2009. MMWR Suppl. 2014;63(5):1‐22. 24941443

[49] Anastos K , Schneider MF , Gange SJ , et al. The association of race, sociodemographic, and behavioral characteristics with response to highly active antiretroviral therapy in women. J Acquir Immune Defic Syndr. 2005;39(5):537‐544. 16044004

[50] Maryland Department of Health . Quick Maryland HIV statistics. Accessed October 31, 2022. https://health.maryland.gov/phpa/OIDEOR/CHSE/pages/statistics.aspx

